# Hyperhomocysteinemia in sudden sensorineural hearing loss: Evidence from a prospective clinical study

**DOI:** 10.1371/journal.pone.0355181

**Published:** 2026-08-06

**Authors:** Murat Akın, Orhan Kemal Kahveci

**Affiliations:** Department of Otorhinolaryngology, Faculty of Medicine, Afyonkarahisar Health Sciences University, Afyonkarahisar, Türkiye; LSU Health Shreveport, UNITED STATES OF AMERICA

## Abstract

**Objective:**

To compare baseline and post-treatment homocysteine levels in patients with sudden sensorineural hearing loss (SSNHL) and to evaluate the potential role of homocysteine in the pathogenesis of the disease as well as its association with hearing recovery.

**Methods:**

A total of 40 patients diagnosed with SSNHL and 40 age- and sex-matched healthy controls were included in this prospective observational study. Serum homocysteine levels were measured at baseline and at the 4–6-week follow-up visit after treatment in the SSNHL group, and once at enrollment in the control group. Hearing thresholds were assessed using pure tone audiometry. Treatment response was classified according to Siegel criteria. Statistical analyses included group comparisons, correlation analyses, and multivariate logistic regression.

**Results:**

Baseline homocysteine levels were significantly higher in the SSNHL group compared with controls. No significant change in homocysteine levels was observed after treatment, although hearing thresholds improved significantly. Homocysteine levels were not significantly associated with hearing recovery. In multivariate logistic regression analysis, baseline homocysteine was independently associated with SSNHL (OR = 1.742, 95% CI: 1.351–2.245, p < 0.001). ROC curve analysis demonstrated good discriminative ability for baseline homocysteine in distinguishing SSNHL patients from controls (AUC = 0.809, 95% CI: 0.712–0.905, p < 0.001).

**Conclusion:**

Homocysteine may play a role in the pathophysiology of SSNHL as a potential vascular risk marker. However, the lack of significant post-treatment reduction and its absence of association with hearing recovery suggest that it may function more as a predisposing factor rather than a prognostic biomarker. Further large-scale prospective studies are needed to evaluate the potential therapeutic implications of homocysteine-lowering strategies in SSNHL.

## Introduction

Sudden sensorineural hearing loss (SSNHL) is an otologic emergency characterized by a hearing loss of at least 30 dB across three consecutive frequencies occurring within 72 hours [1]. It may present with accompanying symptoms such as tinnitus, aural fullness, and vertigo, significantly affecting patients’ quality of life [2]. The incidence of SSNHL ranges from 5 to 27 cases per 100,000 individuals annually, and approximately 90% of cases are classified as idiopathic [3]. Although multiple etiological hypotheses have been proposed, including viral infections, autoimmune mechanisms, and vascular dysfunction, the exact pathophysiology remains incompletely understood [4].

Among these, vascular dysfunction has emerged as one of the most widely accepted mechanisms. The cochlea is particularly vulnerable to ischemic injury due to its lack of collateral blood supply, rendering inner ear perfusion highly sensitive to hemodynamic changes [5]. Consequently, even minor disturbances in cochlear microcirculation, such as endothelial damage or microthrombosis, may trigger sudden hearing loss [6,7]. The abrupt onset of SSNHL resembles clinical events such as transient ischemic attacks, acute myocardial infarction, or retinal vascular occlusion, further supporting the critical role of vascular factors in its pathogenesis [6].

Homocysteine (HCY) is a sulfur-containing amino acid involved in methionine metabolism and plays a key role in biochemical pathways mediated by folic acid and vitamin B12 [8]. Elevated homocysteine levels have been recognized as an independent risk factor for vascular disease due to their association with endothelial dysfunction, increased oxidative stress, platelet activation, and structural alterations of the vascular wall [9,10]. Numerous studies have demonstrated a relationship between hyperhomocysteinemia and peripheral vascular disease, cerebrovascular events, and coronary artery disease [11,12]. In addition to its vascular effects, homocysteine has been implicated in neuronal toxicity through mechanisms such as excitotoxicity, calcium imbalance, DNA damage, and apoptosis [13,14]. Given the delicate microvascular and neural structure of the cochlea, these effects suggest that elevated homocysteine levels may contribute to the development of SSNHL.

In recent years, several clinical studies have investigated the association between SSNHL and homocysteine levels, reporting significantly higher levels in patients compared to healthy controls [15,16]. A comprehensive meta-analysis conducted by Niu et al. further confirmed that homocysteine levels are significantly elevated in SSNHL patients, suggesting a potential predisposing role of hyperhomocysteinemia [17]. However, the majority of these studies have focused solely on baseline homocysteine levels at presentation. Changes in homocysteine levels following treatment and their relationship with clinical recovery remain largely unexplored.

In this context, the present study aims to compare baseline and post-treatment homocysteine levels in patients with SSNHL and to evaluate the potential role of homocysteine in both the pathogenesis and prognosis of the disease. Furthermore, by investigating the relationship between changes in homocysteine levels and hearing recovery, this study seeks to provide additional biochemical evidence supporting vascular mechanisms in SSNHL.

## Materials and methods

### Study design and participants

This study was designed as a prospective observational clinical study. A total of 40 patients diagnosed with sudden sensorineural hearing loss (SSNHL) and 40 age- and sex-matched healthy controls were included. The control group consisted of individuals who presented to the internal medicine department for routine check-ups and had no history of sudden hearing loss, chronic otologic disease, vestibular schwannoma, chronic otitis media, autoimmune disease, ototoxic drug exposure, or active systemic infection. All control participants underwent otologic examination and pure tone audiometric evaluation to exclude clinically relevant hearing impairment.

SSNHL was defined as a sensorineural hearing loss of at least 30 dB affecting three consecutive frequencies within 72 hours. Patients with identifiable causes of hearing loss, including viral infections, autoimmune diseases, ototoxic drug exposure, acoustic trauma, meningitis, vestibular schwannoma, and chronic otitis media, were excluded from the study.

### Data collection

Demographic characteristics and clinical data, including comorbidities such as diabetes mellitus (DM), hypertension (HT), and cardiovascular disease, were recorded for all participants.

In the SSNHL group, hearing thresholds were assessed at presentation using pure tone audiometry. Follow-up audiometric evaluation was performed at the 4–6-week follow-up visit after completion of treatment.

All patients received a standardized treatment protocol consisting of oral methylprednisolone at a dose of 1 mg/kg, followed by gradual tapering at 3-day intervals.

### Laboratory analysis

Venous blood samples were obtained from patients with SSNHL at two time points: at baseline, during initial presentation, and at the follow-up visit, approximately 4–6 weeks after completion of treatment. In the control group, a single venous blood sample was collected at enrollment.

Serum homocysteine levels were measured using an enzyme-linked immunosorbent assay (ELISA) method and expressed in µmol/L.

The data were accessed for research purposes on July 1, 2025. The authors had access to information that could identify individual participants during data collection.

### Audiological evaluation

Pure tone audiometry was performed using standard air- and bone-conduction threshold measurements across conventional audiometric frequencies from 250 Hz to 8 kHz. Additional inter-octave frequencies were assessed when clinically indicated, particularly when there was a threshold difference of 20 dB or more between two adjacent octave frequencies. For statistical analyses, the pure tone average was calculated using thresholds at 0.5, 1, 2, and 4 kHz.

The degree of hearing loss was classified based on the pure tone average (PTA) as follows:

- Normal hearing: 0–25 dB
- Mild hearing loss: 26–40 dB
- Moderate hearing loss: 41–55 dB
- Moderately severe hearing loss: 56–70 dB
- Severe hearing loss: 71–90 dB
- Profound hearing loss: > 90 dB

Treatment response was assessed according to the Siegel classification:

- Type I (Complete recovery): Final PTA ≤ 25 dB
- Type II (Partial recovery): ≥ 15 dB improvement and final PTA between 25–45 dB
- Type III (Slight recovery): ≥ 15 dB improvement but final PTA > 45 dB
- Type IV (No recovery): < 15 dB improvement

Baseline contralateral sensorineural hearing loss was defined as a pure tone average greater than 25 dB in the unaffected ear at initial audiometric evaluation.

### Statistical analysis

All statistical analyses were performed using IBM SPSS Statistics for Windows, Version 26.0. The normality of continuous variables was assessed using the Shapiro–Wilk test and visual inspection of histograms and Q-Q plots. Normally distributed continuous variables were presented as mean ± standard deviation, whereas non-normally distributed variables were presented as median and interquartile range. Categorical variables were presented as numbers and percentages.

For comparisons between two independent groups, the Student’s t-test was used for normally distributed variables and the Mann–Whitney U test was used for non-normally distributed variables. For paired comparisons, the paired samples t-test or Wilcoxon signed-rank test was used, as appropriate. Categorical variables were compared using the Pearson chi-square test. Correlation analyses were performed using Spearman’s correlation analysis. A p-value of <0.05 was considered statistically significant.

Binary logistic regression analysis was performed to identify independent factors associated with SSNHL. Group status was entered as the dependent variable, with SSNHL coded as 1 and control participants coded as 0. Age, sex, diabetes mellitus, hypertension, coronary artery disease, and baseline homocysteine level were included as independent variables. Results were reported as odds ratios with 95% confidence intervals.

Receiver operating characteristic curve analysis was performed to evaluate the discriminative ability of baseline homocysteine levels for SSNHL. The area under the curve, 95% confidence interval, optimal cut-off value, sensitivity, and specificity were calculated. The optimal cut-off value was determined using the Youden index.

### Ethics statement/ IRB approval/ informed consent

This study was conducted in accordance with the principles of the Declaration of Helsinki. Ethical approval was obtained from the Afyonkarahisar Health Sciences University Clinical Research Ethics Committee (Approval No: 2011-KAEK-2; Date: December 1, 2023). Institutional permission was obtained from the Department of Otorhinolaryngology, Faculty of Medicine, Afyonkarahisar Health Sciences University, where the study was conducted. Written informed consent was obtained from all participants prior to enrollment in the study. Patient recruitment was conducted between January 1, 2024, and June 1, 2025.

## Results

### Demographic and clinical characteristics

A total of 40 patients with SSNHL and 40 healthy controls were included in the study. There were no statistically significant differences between the two groups in terms of age (p = 0.686) or sex distribution (p = 0.118). The median age was 54.00 years (IQR: 22.50) in the SSNHL group and 53.00 years (IQR: 28.25) in the control group. Male participants constituted 65.0% of the SSNHL group and 52.5% of the control group.

Similarly, no significant differences were observed between the groups regarding comorbidities, including diabetes mellitus, hypertension, and cardiovascular disease (p = 0.791), indicating comparable baseline clinical characteristics (Table 1).

**Table 1:** Demographic characteristics of the study population.

Variable	Category	SSNHL Group (n = 40)	Control Group (n = 40)	p-value
Sex, n (%)	Male	26 (65.0%)	21 (52.5%)	0.118
	Female	14 (35.0%)	19 (47.5%)	
Age, years	Median [IQR]	54.00 [22.50]	53.00 [28.25]	0.686
Comorbidities, n (%)	None	29 (72.5%)	22 (55.0%)	0.791
	Diabetes mellitus (DM)	2 (5.0%)	6 (15.0%)	
	Hypertension (HT)	1 (2.5%)	2 (5.0%)	
	Coronary artery disease (CAD)	3 (7.5%)	3 (7.5%)	
	DM + HT	4 (10.0%)	6 (15.0%)	
	DM + HT + CAD	1 (2.5%)	1 (2.5%)	

**SSNHL:** sudden sensorineural hearing loss; **IQR:** interquartile range; **DM:** diabetes mellitus; **HT:** hypertensio**n; CAD: c**oronary artery disease. Values are presented as **n (%)** or **median [IQR],** as appropriate. Statistical analyses were performed using the **Pearson chi-square t**est for categorical variables and the **Mann–Whitney U test** for age.

Within the SSNHL group, 26 patients (65.0%) were male and 14 (35.0%) were female (p = 0.058). The distribution of hearing loss laterality was also similar, with 19 patients (47.5%) having right-sided and 21 patients (52.5%) having left-sided hearing loss (p = 0.752) (Table 2).

**Table 2:** Distribution of sex and side of hearing loss in the SSNHL group.

Variable	Category	n (%)	p-value
Sex	Male	26 (65.0%)	0.058
	Female	14 (35.0%)	
Side of hearing loss	Right	19 (47.5%)	0.752
	Left	21 (52.5%)	

**SSNHL:** sudden sensorineural hearing loss.

**Statistical Analysis:** Pearson chi-square test. **p < 0.05** was considered statistically significant.

### Homocysteine levels

Baseline serum homocysteine levels were significantly higher in the SSNHL group compared to the single measurement in controls (median [IQR]: 14.45 [4.00] µmol/L vs. 11.51 [3.48] µmol/L; Mann–Whitney U = 306.000, Z = −4.754, p < 0.001, r = 0.53).

Homocysteine levels measured at the 4–6-week follow-up visit after treatment in the SSNHL group remained significantly higher than the single homocysteine measurement in controls (median [IQR]: 14.35 [4.72] µmol/L vs. 11.51 [3.48]µmol/L; Mann–Whitney U = 328.500, Z = −4.537, p < 0.001, r = 0.51). However, follow-up homocysteine levels in the SSNHL group did not show a statistically significant change compared to baseline values (14.45 [4.00] µmol/L vs. 14.35 [4.72] µmol/L; Wilcoxon signed-rank test, Z = −0.215, p = 0.830, r = 0.03) (Table 3).

**Table 3:** Comparison of homocysteine levels between the SSNHL and control groups.

Parameter	SSNHL Group Median [IQR]	Control Group Median [IQR]	Test statistic	p-value	Effect size r
Baseline homocysteine	14.45 [4.00]	11.51 [3.48]	U = 306.000, Z = −4.754	<0.001	0.53
Post-treatment homocysteine	14.35 [4.72]	11.51 [3.48]*	U = 328.500, Z = −4.537	<0.001	0.51
Baseline vs post-treatment homocysteine	14.45 [4.00] vs. 14.35 [4.72]	—	Z = −0.215	0.830	0.03

SSNHL: Sudden sensorineural hearing loss; IQR: interquartile range. Values are presented as median [IQR]. Statistical analyses were performed using the Mann–Whitney U test for between-group comparisons and the Wilcoxon signed-rank test for within-group comparisons. Effect size was calculated as r = Z/√N. Absolute r values are reported. *Control group values represent a single homocysteine measurement, as no treatment was administered to the control group.

### Hearing outcomes

Hearing loss severity in the SSNHL group was distributed across a wide range at baseline, from mild to profound levels. Following treatment, a statistically significant improvement in hearing thresholds was observed (p < 0.001).

Before treatment, the distribution of hearing loss was as follows: mild (27.5%), moderate (20.0%), moderately severe (12.5%), severe (27.5%), and profound (12.5%).

At baseline, clinically significant contralateral sensorineural hearing loss was present in 9 patients (22.5%).

After treatment, 35.0% of patients achieved normal hearing, while the proportions of moderate and severe hearing loss decreased substantially. No patients remained in the profound hearing loss category after treatment (Table 4).

**Table 4:** Comparison of hearing loss severity before and after treatment in the SSNHL group.

Hearing Level (PTA Classification)	Pre-treatment n (%)	Post-treatment n (%)	p-value
Normal (0–25 dB)	0 (0.0%)	14 (35.0%)	<0.001
Mild (26–40 dB)	11 (27.5%)	10 (25.0%)	
Moderate (41–55 dB)	8 (20.0%)	4 (10.0%)	
Moderately severe (56–70 dB)	5 (12.5%)	9 (22.5%)	
Severe (71–90 dB)	11 (27.5%)	3 (7.5%)	
Profound (>90 dB)	5 (12.5%)	0 (0.0%)	

**PTA:** pure tone average; **SSNHL:** sudden sensorineural hearing loss.

**Statistical Analysis:** Wilcoxon signed-rank test. **p < 0.05** was considered statistically significant.

### Correlation analyses

Baseline homocysteine levels were strongly correlated with post-treatment homocysteine levels in the SSNHL group (Spearman’s rho = 0.876, p < 0.001), suggesting that individual homocysteine levels remained relatively stable over time. However, no significant overall change was observed between baseline and post-treatment homocysteine levels according to the Wilcoxon signed-rank test.

Similarly, pre-treatment PTA was positively correlated with post-treatment PTA (Spearman’s rho = 0.628, p < 0.001). However, baseline homocysteine levels were not significantly correlated with pre-treatment PTA (Spearman’s rho = 0.261, p = 0.104) or post-treatment PTA (Spearman’s rho = 0.280, p = 0.080). Similarly, post-treatment homocysteine levels were not significantly correlated with pre-treatment PTA (Spearman’s rho = 0.085, p = 0.601) or post-treatment PTA (Spearman’s rho = 0.227, p = 0.159). These findings indicate that homocysteine levels were not significantly associated with hearing outcomes (Table 5).

**Table 5:** Correlation analysis between homocysteine levels and hearing outcomes.

Variable pair	Spearman’s rho	p-value
Baseline homocysteine – Post-treatment homocysteine	0.876	<0.001
Baseline homocysteine – Pre-treatment PTA	0.261	0.104
Baseline homocysteine – Post-treatment PTA	0.280	0.080
Post-treatment homocysteine – Pre-treatment PTA	0.085	0.601
Post-treatment homocysteine – Post-treatment PTA	0.227	0.159
Pre-treatment PTA – Post-treatment PTA	0.628	<0.001

**PTA:** pure tone average. **Statistical Analysis:** Spearman correlation analysis. Correlation analyses were performed within the SSNHL group. **p < 0.05** was considered statistically significant.

### Analysis according to Siegel classification

When evaluated according to the Siegel classification, baseline homocysteine levels did not differ significantly among recovery groups (p = 0.082).

Similarly, post-treatment homocysteine levels were not significantly associated with treatment response (p = 0.219) (Table 6).

**Table 6:** Comparison of homocysteine levels according to Siegel recovery classification.

Siegel Classification	Baseline Homocysteine Median [IQR] (µmol/L)	Post-treatment Homocysteine Median [IQR] (µmol/L)
Type I – Complete recovery	12.70 [5.50]	12.80 [7.31]
Type II – Partial recovery	16.10 [4.98]	15.15 [4.97]
Type III – Slight recovery	13.90 [NA]	12.60 [NA]
Type IV – No recovery	14.85 [3.75]	14.85 [1.90]

**IQR:** interquartile range; **NA:** not applicable due to the small number of observations. Values are presented as median [IQR]. Statistical Analysis: Kruskal–Wallis test (Baseline: p = 0.082, Post-treatment: p = 0.219)

Spearman correlation analysis demonstrated no statistically significant association between Siegel recovery classification and baseline homocysteine levels (rho = 0.148, p = 0.363) or post-treatment homocysteine levels (rho = 0.158, p = 0.329). These results suggest that homocysteine levels were not significantly related to treatment response according to the Siegel classification (Table 7).

**Table 7:** Correlation between homocysteine levels and Siegel recovery classification.

Variable pair	Spearman’s rho	p-value
Baseline homocysteine – Post-treatment homocysteine	0.876	<0.001
Baseline homocysteine – Siegel classification	0.148	0.363
Post-treatment homocysteine – Siegel classification	0.158	0.329

**Statistical Analysis:** Spearman correlation analysis. Siegel classification was evaluated as an ordinal recovery variable. Correlation analyses were performed within the SSNHL group. **p < 0.05** was considered statistically significant.

### Logistic regression analysis

Binary logistic regression analysis was performed to identify independent factors associated with SSNHL. Age, sex, diabetes mellitus, hypertension, coronary artery disease, and baseline homocysteine level were included in the model. Among these variables, only baseline homocysteine level was independently associated with SSNHL. Each 1 µmol/L increase in baseline homocysteine level was associated with a 1.742-fold increase in the odds of SSNHL (OR = 1.742, 95% CI: 1.351–2.245, p < 0.001). Age, sex, diabetes mellitus, hypertension, and coronary artery disease were not independently associated with SSNHL in the multivariate model (Table 8).

**Table 8:** Multivariate logistic regression analysis of factors associated with SSNHL.

Variable	B	SE	Wald	OR	95% CI	p-value
Sex	0.417	0.624	0.446	1.517	0.446–5.155	0.504
Age	0.010	0.022	0.207	1.010	0.968–1.054	0.649
Baseline homocysteine	0.555	0.130	18.341	1.742	1.351–2.245	<0.001
Diabetes mellitus	−1.216	0.878	1.921	0.296	0.053–1.655	0.166
Hypertension	−0.521	0.939	0.307	0.594	0.094–3.744	0.579
Coronary artery disease	−1.517	0.987	2.363	0.219	0.032–1.518	0.124

SSNHL: sudden sensorineural hearing loss; SE: standard error; OR: odds ratio; CI: confidence interval. Statistical Analysis: Binary logistic regression analysis using the Enter method. Sex, diabetes mellitus, hypertension, and coronary artery disease were entered as categorical variables.

### ROC curve analysis

**Fig 1:**
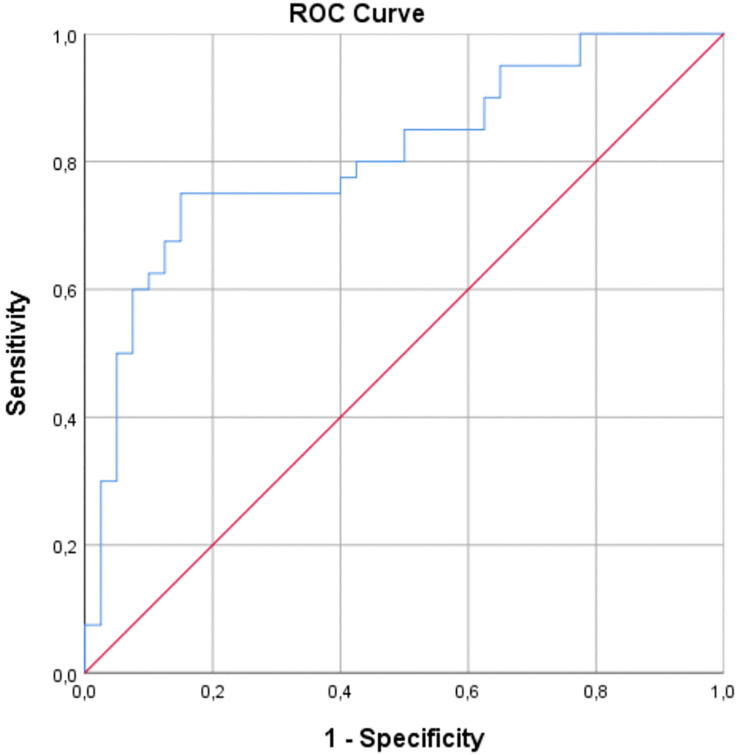
Receiver operating characteristic curve of baseline homocysteine levels for distinguishing patients with sudden sensorineural hearing loss from controls. The area under the curve was 0.809 (95% CI: 0.712–0.905, p < 0.001).

Receiver operating characteristic curve analysis was performed to evaluate the discriminative ability of baseline homocysteine levels for distinguishing patients with SSNHL from controls. Baseline homocysteine demonstrated an area under the curve of 0.809 (95% CI: 0.712–0.905, p < 0.001), indicating good discriminative ability. The optimal cut-off value was 12.60 µmol/L, with a sensitivity of 75.0%, specificity of 85.0%, and Youden index of 0.600 (Table 9 and Fig 1).

**Table 9:** ROC curve analysis of baseline homocysteine for distinguishing SSNHL from controls.

Variable	AUC	SE	95% CI	p-value	Cut-off value	Sensitivity	Specificity	Youden index
Baseline homocysteine	0.809	0.049	0.712–0.905	<0.001	12.60 µmol/L	75.0%	85.0%	0,600

**AUC:** Area under the curve; **SE:** Standard error; **CI:** confidence interval; **SSNHL:** sudden sensorineural hearing loss. The optimal cut-off value was determined using the Youden index.

## Discussion

Sudden sensorineural hearing loss (SSNHL) is a multifactorial disorder with a complex and not yet fully elucidated pathophysiology. Epidemiological studies have shown that SSNHL most commonly occurs between the ages of 30 and 50 years, affects both sexes with similar incidence rates, and typically presents with a relatively symmetrical distribution in terms of laterality [3]. In the present study, the age distribution, sex ratio, and laterality of hearing loss were highly consistent with the data reported in the literature, supporting the notion that the epidemiological profile of SSNHL is relatively homogeneous.

The etiology of SSNHL is multifactorial, with vascular mechanisms increasingly emphasized in recent years. The absence of collateral circulation in the cochlea renders it highly susceptible to microvascular perfusion disturbances, which may result in sudden and clinically significant hearing impairment [18–20]. Although several studies have reported that systemic vascular risk factors such as hypertension, diabetes mellitus, and coronary artery disease may contribute to the development of idiopathic SSNHL, conflicting findings have also been published [6,21,22]. In the present study, diabetes mellitus, hypertension, and coronary artery disease were included in the multivariate logistic regression model as potential vascular comorbidities. However, none of these comorbidities showed an independent association with SSNHL. This finding may be partly explained by the comparable distribution of vascular comorbidities between the SSNHL and control groups, as well as the relatively small sample size. Because the control group was selected to be comparable with the SSNHL group in terms of age and sex, the present study was not primarily designed to evaluate age or sex as independent risk factors for SSNHL. Therefore, the absence of a significant association should not be interpreted as excluding the potential role of systemic vascular risk factors in SSNHL. Rather, our findings suggest that baseline homocysteine may be more closely associated with SSNHL than conventional vascular comorbidities in this study population.

One of the most striking findings of this study is the significantly elevated homocysteine levels observed in patients with SSNHL compared to healthy controls. This finding is consistent with multiple clinical studies reporting higher homocysteine levels in SSNHL patients [15,16]. Furthermore, our multivariate logistic regression analysis demonstrated that baseline homocysteine level is an independent risk factor for SSNHL. The observation that each 1 µmol/L increase in homocysteine level increases the risk of SSNHL by 1.74-fold (OR = 1.742) further supports the potential role of this biomarker in the vascular pathophysiology of the disease. Similarly, the comprehensive meta-analysis conducted by Niu et al. demonstrated significantly higher homocysteine levels in SSNHL patients compared to controls and suggested a potential predisposing role for hyperhomocysteinemia [17].

In addition to the group comparison and logistic regression findings, ROC curve analysis further supported the discriminative value of baseline homocysteine levels. Baseline homocysteine demonstrated good ability to distinguish patients with SSNHL from controls, with an AUC of 0.809. The optimal cut-off value of 12.60 µmol/L provided a sensitivity of 75.0% and specificity of 85.0%. These findings suggest that elevated baseline homocysteine may have potential diagnostic relevance as a vascular biomarker in SSNHL. However, this cut-off value should be interpreted cautiously and requires validation in larger, independent cohorts before being applied in clinical practice.

The biological mechanisms underlying this association are well supported by existing evidence. Homocysteine is known to impair vascular endothelial function, increase oxidative stress, and promote thrombotic activity, ultimately leading to structural alterations in the vascular wall [9,10]. Recent evidence has also suggested that coagulation dysfunction, elevated homocysteine levels, and serum oxidative stress markers may be clinically relevant in sudden deafness [23]. When these mechanisms are considered in the context of the cochlear microvascular system, disruption of inner ear perfusion may occur, resulting in sudden hearing loss. Indeed, several studies have demonstrated that elevated homocysteine levels negatively affect microvascular circulation and are consistent with the vascular hypothesis of SSNHL [23,24]. Taken together, these findings indicate that hyperhomocysteinemia may represent a significant biochemical marker supporting the vascular origin of SSNHL.A major strength of the present study is that, unlike most previous studies, it evaluates not only baseline homocysteine levels but also post-treatment levels. The majority of studies in the literature have relied on single time-point measurements, and the effect of treatment on homocysteine levels has not been adequately investigated. In our study, the absence of a significant change in homocysteine levels following treatment suggests that homocysteine may function primarily as a predisposing or chronic risk factor rather than a dynamic marker of disease activity.

In addition to its vascular effects, homocysteine is known to contribute to neuronal toxicity through oxidative stress and free radical formation [13,25,26]. Elevated homocysteine levels can trigger glutamate-mediated excitotoxicity, increase the production of reactive oxygen species, and lead to structural damage in both spiral ganglion neurons and supporting cells of the cochlea [13,14]. From a pathophysiological perspective, reducing homocysteine levels may therefore not only improve endothelial function but also decrease oxidative stress and neuronal damage, potentially contributing to hearing recovery. Accordingly, homocysteine-lowering treatments, such as folate and vitamin B12 supplementation, may represent a complementary therapeutic strategy in SSNHL by improving cochlear microcirculation and reducing oxidative stress burden [27,28].

When the relationship between homocysteine levels and hearing outcomes was evaluated, no significant correlation was found between baseline or post-treatment homocysteine levels and hearing thresholds. Similarly, analysis based on the Siegel classification revealed no significant association between homocysteine levels and the degree of recovery. These findings suggest that homocysteine may not be a reliable prognostic biomarker for treatment response. Supporting our findings, Unal et al. reported that although homocysteine levels were significantly higher in patients with acute ischemic stroke compared to controls, they were not predictive of disease severity, functional disability, or prognosis [29]. In contrast, the strong correlation observed between pre-treatment and post-treatment hearing levels confirms the effectiveness of steroid therapy and is consistent with the current literature [30].

The most important contribution of this study lies in the simultaneous evaluation of both baseline and post-treatment homocysteine levels. This approach provides a more comprehensive understanding of the role of homocysteine in both the onset and recovery phases of SSNHL. However, several limitations should be acknowledged, including the relatively small sample size, heterogeneity in subgroup distribution, and the absence of long-term follow-up data.

Taken together, these findings highlight that the pathogenesis of SSNHL cannot be attributed to a single mechanism but rather reflects a dynamic interaction of vascular dysfunction, oxidative stress, neuronal vulnerability, and immunological processes. The cochlear microcirculation appears to be highly sensitive to systemic vascular biomarkers, and even subtle endothelial dysfunction may have profound effects on auditory function. In this context, the dual vascular and neurotoxic effects of homocysteine further strengthen its relevance as a biomarker in SSNHL.

From a clinical perspective, the course and treatment response of SSNHL may depend not only on audiometric findings but also on the patient’s vascular and metabolic profile. Future large-scale, prospective, and multicenter studies are needed to clarify the prognostic significance of homocysteine and other vascular biomarkers, as well as their potential role as therapeutic targets.

Overall, our findings demonstrate that homocysteine levels are elevated in patients with SSNHL and remain elevated during the recovery period, suggesting a potential role in disease pathogenesis. These results are consistent with previous meta-analyses and clinical studies indicating that hyperhomocysteinemia may be a risk factor for SSNHL. Further large-scale prospective studies are required to better define the clinical utility of this biomarker and its potential role in guiding therapeutic strategies.

## Limitations

The present study has several limitations. First, the relatively small sample size and single-center design may limit the generalizability of the findings. Second, homocysteine levels were evaluated at only a single post-treatment time point, which may not fully reflect long-term biochemical changes. In addition, clinically significant baseline contralateral sensorineural hearing loss was observed in 9 patients (22.5%). As the Siegel classification may be influenced by pre-existing contralateral hearing loss, this factor should be considered when interpreting treatment response. However, recovery outcomes were evaluated using standardized audiometric criteria. Despite these limitations, the prospective design of the study and the simultaneous evaluation of both baseline and post-treatment homocysteine levels provide a significant contribution to the existing literature.

## Conclusion

This study demonstrates that homocysteine levels are significantly elevated in patients with sudden sensorineural hearing loss (SSNHL) compared to healthy controls, suggesting that hyperhomocysteinemia may play an important role in the pathogenesis of the disease.

The comparison of baseline and post-treatment homocysteine levels indicates that, although this biomarker appears to be involved in the onset of SSNHL, its changes during the treatment process are limited. The absence of a significant decrease in homocysteine levels despite substantial improvement in hearing thresholds suggests that homocysteine may function more as a predisposing factor rather than a dynamic marker of treatment response.

Considering the effects of homocysteine on vascular endothelium, oxidative stress, and neuronal toxicity, this molecule may contribute to cochlear microcirculatory impairment and neural damage, thereby providing a plausible biological explanation for SSNHL.

In conclusion, homocysteine measurement may serve as a valuable biomarker in SSNHL from both diagnostic and pathophysiological perspectives. Future large-scale prospective studies evaluating the impact of homocysteine-lowering therapies, such as folate, vitamin B12, and antioxidant supplementation, on SSNHL prognosis will be crucial for determining the clinical applicability of this biochemical target.
