# Supplementary material for: Hyperhomocysteinemia in sudden sensorineural hearing loss: Evidence from a prospective clinical study
Source: PLoS One. 2026 Aug 6;21(8):e0355181. doi: 10.1371/journal.pone.0355181 (PMC13446708; doi:10.1371/journal.pone.0355181)
Supplement: Supplementary file 1 [file pone.0355181.s002.docx]

Response to Reviewers

Manuscript ID: PONE-D-26-22703
Title: Hyperhomocysteinemia in sudden sensorineural hearing loss: Evidence from a prospective clinical study

Dear Editor and Reviewers,

We would like to thank the Academic Editor and the reviewers for their careful evaluation of our manuscript and for their constructive comments. We have revised the manuscript accordingly and believe that the changes have substantially improved the clarity, methodological transparency, and scientific rigor of the study. All changes have been incorporated into the revised manuscript. Our point-by-point responses are provided below.

Editor / Reviewer comment 1:
The Data Availability Statement should not state that data are available from the corresponding author. A non-author institutional contact should be provided.

Response:
Thank you for this important comment. We have revised the Data Availability Statement accordingly. The revised statement now clarifies that the data are not publicly available due to ethical and institutional restrictions concerning participant confidentiality. We have removed the statement indicating that data are available from the corresponding author and instead provided an institutional contact point, namely the Afyonkarahisar Health Sciences University Clinical Research Ethics Committee or the relevant institutional data access body, for researchers who meet the criteria for access to confidential data.

Revised manuscript text:
“The data underlying the findings of this study are not publicly available due to ethical and institutional restrictions concerning participant confidentiality. Data may be made available upon reasonable request from the Afyonkarahisar Health Sciences University Clinical Research Ethics Committee (contact number: +90 272 246 33 01) or the relevant institutional data access body, for researchers who meet the criteria for access to confidential data.”

Reviewer comment 2:
The selection criteria and characteristics of the control group should be clarified.

Response:
Thank you for this comment. We have revised the Materials and methods section to provide a clearer definition of the control group. We now specify that the control participants were age- and sex-matched individuals who presented to the internal medicine department for routine check-ups and had no history of sudden hearing loss, chronic otologic disease, vestibular schwannoma, chronic otitis media, autoimmune disease, ototoxic drug exposure, or active systemic infection. We also clarified that all control participants underwent otologic examination and pure tone audiometric evaluation to exclude clinically relevant hearing impairment.

Reviewer comment 3:
The audiometric methodology should be revised to comply with standard audiometric procedures. Standard audiometry includes assessment from 250 Hz to 8 kHz and inter-octave frequencies when clinically indicated.

Response:
Thank you for this important methodological comment. We agree with the reviewer and have revised the Audiological evaluation section. The revised text now states that pure tone audiometry was performed using standard air- and bone-conduction threshold measurements across conventional audiometric frequencies from 250 Hz to 8 kHz. We also clarified that additional inter-octave frequencies were assessed when clinically indicated, particularly when there was a threshold difference of 20 dB or more between two adjacent octave frequencies. For statistical analyses, the pure tone average was calculated using thresholds at 0.5, 1, 2, and 4 kHz.

Reviewer comment 4:
The timing of the second blood sample should be clearly stated.

Response:
Thank you for this comment. We have clarified the timing of the second venous blood sample in both the Abstract and the Laboratory analysis section. The revised manuscript now states that serum homocysteine levels were measured at baseline and at the 4–6-week follow-up visit after treatment in the SSNHL group, and once at enrollment in the control group.

Reviewer comment 5:
The term “post-treatment” should not be used for the control group because controls did not receive treatment.

Response:
Thank you for this important observation. We agree that this wording could be misleading. We have revised the Results section and Table 3 to clarify that the control group had only a single homocysteine measurement. We also added a table footnote stating that control group values represent a single homocysteine measurement because no treatment was administered to the control group.

Reviewer comment 6:
The statistical reporting should follow AMA-style reporting standards, including appropriate descriptive statistics, test statistics, p-values, and effect sizes.

Response:
Thank you for this valuable suggestion. We have revised the Statistical analysis section and the Results section accordingly. We assessed normality using the Shapiro–Wilk test and visual inspection of histograms and Q-Q plots. Normally distributed continuous variables were described as mean ± standard deviation, whereas non-normally distributed variables were presented as median and interquartile range. Categorical variables were presented as numbers and percentages. We also added the relevant test statistics and effect sizes for significant findings, including Mann–Whitney U values, Z values, p-values, and r effect sizes where appropriate. Logistic regression results are reported as odds ratios with 95% confidence intervals.

Reviewer comment 7:
Effect sizes should be reported for significant findings.

Response:
Thank you for this comment. We have added effect sizes for the significant non-parametric comparisons. For Mann–Whitney U and Wilcoxon signed-rank tests, effect size was calculated as r = Z/√N. These values are now presented in Table 3 and described in the Results section. For logistic regression, odds ratios with 95% confidence intervals are reported, and for ROC analysis, the area under the curve with 95% confidence interval is presented.

Reviewer comment 8:
Table 5 contains values that appear unusually high and may represent incorrectly formatted PTA values.

Response:
Thank you for pointing this out. We reviewed Table 5 and found that the values represented Spearman correlation coefficients, not PTA values. However, due to formatting, several correlation coefficients were displayed without decimal points, which may have caused confusion. We have corrected the table and now present each variable pair separately with Spearman’s rho and the corresponding p-value. The corrected values are reported as 0.261, 0.280, 0.085, and 0.227 rather than 261, 280, 85, and 227.

Reviewer comment 9:
Table 8 should summarize all variables included in the logistic regression model, not only the significant predictor.

Response:
Thank you for this comment. We have revised Table 8 to include all variables entered into the multivariate logistic regression model, including age, sex, diabetes mellitus, hypertension, coronary artery disease, and baseline homocysteine level. The revised table reports B, standard error, Wald statistic, odds ratio, 95% confidence interval, and p-value for each variable. The Results section has also been revised accordingly.

Reviewer comment 10:
The SPSS version should be specified.

Response:
Thank you for this comment. We have revised the Statistical analysis section to specify the software used. The revised manuscript now states: “All statistical analyses were performed using IBM SPSS Statistics for Windows, Version 26.0.”

Reviewer comment 11:
Because the control group was age- and sex-matched, the manuscript should avoid interpreting age and sex as independent risk factors.

Response:
Thank you for this important methodological point. We have revised the Discussion section to clarify that the study was not primarily designed to evaluate age or sex as independent risk factors for SSNHL because the control group was selected to be comparable with the SSNHL group in terms of age and sex. We also clarified that the absence of a significant association should not be interpreted as excluding the potential contribution of systemic vascular risk factors in SSNHL.

Reviewer comment 12:
ROC curve analysis should be performed for baseline homocysteine levels.

Response:
Thank you for this suggestion. We performed ROC curve analysis to evaluate the discriminative ability of baseline homocysteine levels for distinguishing SSNHL patients from controls. Baseline homocysteine demonstrated good discriminative ability, with an AUC of 0.809 (95% CI: 0.712–0.905, p < 0.001). The optimal cut-off value was 12.60 µmol/L, with a sensitivity of 75.0%, specificity of 85.0%, and Youden index of 0.600. These results have been added to the Results section, Table 9, and Fig 1. We also discussed the ROC findings in the Discussion section and emphasized that the cut-off value should be validated in larger independent cohorts before clinical application.

Reviewer comment 13:
The Siegel classification may be limited when baseline contralateral hearing loss is present. How many patients had sensorineural hearing loss in the contralateral ear?

Response:
Thank you for this important comment. We reviewed the baseline audiometric data of the unaffected contralateral ear. Clinically significant baseline contralateral sensorineural hearing loss was defined as a pure tone average greater than 25 dB in the unaffected ear at initial audiometric evaluation. Based on this definition, clinically significant baseline contralateral sensorineural hearing loss was present in 9 patients (22.5%). This information has been added to the Results section. We also acknowledged this issue in the Limitations section, noting that the Siegel classification may be influenced by pre-existing contralateral hearing loss and that this factor should be considered when interpreting treatment response.

Reviewer comment 14:
Please disclose whether artificial intelligence tools or large language models were used during manuscript preparation.

Response:
Thank you for this comment. We have added an Artificial Intelligence Disclosure section to the manuscript. The revised disclosure states that an AI-based language model was used only for language editing and improving clarity. No AI tool was used for study design, data collection, data analysis, interpretation of results, or generation of scientific conclusions. The authors reviewed and approved the final content and take full responsibility for the integrity and accuracy of the manuscript.

Reviewer comment 15:
Please consider adding recent literature relevant to homocysteine, coagulation function, oxidative stress, and sudden deafness.

Response:
Thank you for this helpful suggestion. We have added the recent study by Wang et al. concerning the relationship between coagulation function, homocysteine, serum oxidative stress, and sudden deafness. This reference has been incorporated into the Discussion section to strengthen the biological rationale linking homocysteine, oxidative stress, coagulation dysfunction, and cochlear microvascular impairment in SSNHL.

Reviewer comment 16:
The manuscript should be revised according to PLOS ONE formatting requirements.

Response:
Thank you for this comment. We revised the manuscript formatting in accordance with PLOS ONE style. The title has been changed to sentence case, section numbering has been removed, in-text citations have been changed from parentheses to square brackets, tables have been simplified, and the ROC curve has been presented as Fig 1 with an appropriate caption. We also revised the title page and declarations section to improve consistency with journal formatting requirements.

Reviewer comment 17:
Please ensure that the manuscript includes appropriate ethical approval and informed consent information.

Response:
Thank you for this comment. We have revised the Ethics Statement / IRB Approval / Informed Consent section. The manuscript now states that the study was conducted in accordance with the Declaration of Helsinki, that ethical approval was obtained from the Afyonkarahisar Health Sciences University Clinical Research Ethics Committee (Approval No: 2011-KAEK-2; Date: December 1, 2023), that institutional permission was obtained from the Department of Otorhinolaryngology, Faculty of Medicine, Afyonkarahisar Health Sciences University, and that written informed consent was obtained from all participants prior to enrollment.

We thank the Editor and reviewers again for their constructive comments. We believe that the revised manuscript now addresses all concerns raised during the review process.

Sincerely,

Murat Akın
On behalf of all authors
