# Supplementary material for: Hyperhomocysteinemia in sudden sensorineural hearing loss: Evidence from a prospective clinical study
Source: PLoS One. 2026 Aug 6;21(8):e0355181. doi: 10.1371/journal.pone.0355181 (PMC13446708; doi:10.1371/journal.pone.0355181)
Supplement: Peer review history

# Peer review: Decision Letter 0

**DOI:** 10.1371/journal.pone.0355181.r001

**Authors:** Gauri Mankekar

19 Jun 2026

PONE-D-26-22703

Hyperhomocysteinemia in Sudden Sensorineural Hearing Loss: Evidence From a Prospective Clinical Study

PLOS One

Dear Dr. Akın,

Thank you for submitting your manuscript to PLOS ONE. After careful consideration, we feel that it has merit but does not fully meet PLOS ONE’s publication criteria as it currently stands. Therefore, we invite you to submit a revised version of the manuscript that addresses the points raised during the review process.

==============================

**ACADEMIC EDITOR:**  Please see the reviewer's comments and respond to their comments. Also revise the manuscript appropriately per their suggestions before resubmitting

==============================

Please submit your revised manuscript by Jul 19 2026 11:59PM. If you will need more time than this to complete your revisions, please reply to this message or contact the journal office at plosone@plos.org. When you're ready to submit your revision, log on to https://www.editorialmanager.com/pone/ and select the 'Submissions Needing Revision' folder to locate your manuscript file.

Please include the following items when submitting your revised manuscript:

- A letter that responds to each point raised by the academic editor and reviewer(s). You should upload this letter as a separate file labeled 'Response to Reviewers'.
- A marked-up copy of your manuscript that highlights changes made to the original version. You should upload this as a separate file labeled 'Revised Manuscript with Track Changes'.
- An unmarked version of your revised paper without tracked changes. You should upload this as a separate file labeled 'Manuscript'.

If you would like to make changes to your financial disclosure, please include your updated statement in your cover letter. Guidelines for resubmitting your figure files are available below the reviewer comments at the end of this letter.

If applicable, we recommend that you deposit your laboratory protocols in protocols.io to enhance the reproducibility of your results. Protocols.io assigns your protocol its own identifier (DOI) so that it can be cited independently in the future. For instructions see: https://journals.plos.org/plosone/s/submission-guidelines#loc-laboratory-protocols. Additionally, PLOS ONE offers an option for publishing peer-reviewed Lab Protocol articles, which describe protocols hosted on protocols.io. Read more information on sharing protocols at https://plos.org/protocols?utm_medium=editorial-email&utm_source=authorletters&utm_campaign=protocols.

As the corresponding author, your ORCID iD is verified in the submission system and will appear in the published article. PLOS supports the use of ORCID, and we encourage all coauthors to register for an ORCID iD and use it as well. Please encourage your coauthors to verify their ORCID iD within the submission system before final acceptance, as unverified ORCID iDs will not appear in the published article. *Only*  the individual author can complete the verification step; PLOS staff *cannot*  verify ORCID iDs on behalf of authors.

We look forward to receiving your revised manuscript.

Kind regards,

Gauri Mankekar, MD,PhD,FACS

Academic Editor

PLOS One

**Journal Requirements:**

When submitting your revision, we need you to address these additional requirements.

1. Please ensure that your manuscript meets PLOS ONE's style requirements, including those for file naming. The PLOS ONE style templates can be found at

https://journals.plos.org/plosone/s/file?id=wjVg/PLOSOne_formatting_sample_main_body.pdf and

https://journals.plos.org/plosone/s/file?id=ba62/PLOSOne_formatting_sample_title_authors_affiliations.pdf

2. In the online submission form you indicate that your data is not available for proprietary reasons and have provided a contact point for accessing this data. Please note that your current contact point is a co-author on this manuscript. According to our Data Policy, the contact point must not be an author on the manuscript and must be an institutional contact, ideally not an individual. Please revise your data statement to a non-author institutional point of contact, such as a data access or ethics committee, and send this to us via return email. Please also include contact information for the third party organization, and please include the full citation of where the data can be found.

3. If the reviewer comments include a recommendation to cite specific previously published works, please review and evaluate these publications to determine whether they are relevant and should be cited. There is no requirement to cite these works unless the editor has indicated otherwise.

4. Please review your reference list to ensure that it is complete and correct. If you have cited papers that have been retracted, please include the rationale for doing so in the manuscript text, or remove these references and replace them with relevant current references. Any changes to the reference list should be mentioned in the rebuttal letter that accompanies your revised manuscript. If you need to cite a retracted article, indicate the article’s retracted status in the References list and also include a citation and full reference for the retraction notice.

[Note: HTML markup is below. Please do not edit.]

Reviewers' comments:

Reviewer's Responses to Questions

**Comments to the Author**

1. Is the manuscript technically sound, and do the data support the conclusions?

The manuscript must describe a technically sound piece of scientific research with data that supports the conclusions. Experiments must have been conducted rigorously, with appropriate controls, replication, and sample sizes. The conclusions must be drawn appropriately based on the data presented.

Reviewer #1: Yes

Reviewer #2: Yes

**********

2. Has the statistical analysis been performed appropriately and rigorously?

Reviewer #1: Yes

Reviewer #2: Yes

**********

3. Have the authors made all data underlying the findings in their manuscript fully available?

The PLOS Data policy requires authors to make all data underlying the findings described in their manuscript fully available without restriction, with rare exception (please refer to the Data Availability Statement in the manuscript PDF file). The data should be provided as part of the manuscript or its supporting information, or deposited to a public repository. For example, in addition to summary statistics, the data points behind means, medians and variance measures should be available. If there are restrictions on publicly sharing data—e.g. participant privacy or use of data from a third party—those must be specified.

Reviewer #1: Yes

Reviewer #2: No

**********

4. Is the manuscript presented in an intelligible fashion and written in standard English?

PLOS ONE does not copyedit accepted manuscripts, so the language in submitted articles must be clear, correct, and unambiguous. Any typographical or grammatical errors should be corrected at revision, so please note any specific errors here.

Reviewer #1: Yes

Reviewer #2: Yes

**********

5. Review Comments to the Author

Please use the space provided to explain your answers to the questions above. You may also include additional comments for the author, including concerns about dual publication, research ethics, or publication ethics. (Please upload your review as an attachment if it exceeds 20,000 characters)

Reviewer #1: Reviewer Comments

1. The statement about SSNHL on page 3, lines 34–35 appears to be repetitive and may be removed.

2. The description on page 4, lines 15–16 does not follow the standard audiometric procedure. Standard audiometry includes hearing threshold assessment from 250 Hz to 8 kHz, covering all octave frequencies and mid-octaves when the threshold difference between two consecutive octaves is 20 dB or more. Please justify this methodology.

3. Table 3 indicates “post-treatment” for the control group, which is misleading because no treatment was administered to the control group. Please revise the table wording accordingly.

4. In Table 5, the pre- and post-PTA values appear unusually high. Please correct these values or provide appropriate justification.

5. Table 8 should summarize all variables other than the significant predictor variable.

6. Please correct the error on page 4, line 30 regarding SPSS software and mention the exact version used.

7. Please report the effect size for all significant findings in the Results section and include the corresponding test statistics along with the p-values.

Reviewer #2: This is a prospective study evaluating homocysteine levels in patients with sudden sensorineural loss versus controls. The authors use two time points to evaluate homocysteine—once at diagnosis and once after treatment—and they find that there was no change in homocysteine after therapy. This is an interesting observation and adds to recent work around this biomarker.

I’m unclear how the authors chose the control group—did they come from an ENT clinic or some other means? What degree of hearing loss do they have? While the authors claim that the control group is age and sex matched with the study group, there appears to be a difference, albeit not statistically significant, between the sex distribution of the control and study groups. Additionally, if the authors claim there is a sex and age match, they can’t also claim that these “were not identified as independent risk factors for SSNHL” (para 2 of discussion).

Consider avoiding “significant” throughout the manuscript if something isn’t “statistically significant.”

Please consider using AMA reporting standards for reporting statistical data. The authors report means and standard deviations for non-parametric data, when they ought to use medians and IQRs. The authors also use asterisks for describing the statistical test used in their tables, but this is frequently the notation used for statistical significance. This is confusing throughout the manuscript. I’m also not clear why a ROC curve wasn’t used for evaluating SSNHL risk with homocysteine levels (para 3.6).

The authors reference older work on this topic, and newer work is available to incorporate (eg PMID 41922431).

How soon after treatment was the second lab draw?

Please include the version of SPSS used. “XX” appears to be a placeholder.

The Siegel classification is frequently used to describe recovery of SSNHL, but it often fails if there’s baseline hearing loss. How many patients have SNHL in the contralateral ear?

Please also disclose in the manuscript if or if not AI/LLMs were used in the study design, literature review, translation assistance, or manuscript preparation.

**********

6. PLOS authors have the option to publish the peer review history of their article (what does this mean?). If published, this will include your full peer review and any attached files.

If you choose “no”, your identity will remain anonymous but your review may still be made public.

**Do you want your identity to be public for this peer review?**  For information about this choice, including consent withdrawal, please see our Privacy Policy.

Reviewer #1: **Yes:**  Jithin Raj Balan

Reviewer #2: No

**********

[NOTE: If reviewer comments were submitted as an attachment file, they will be attached to this email and accessible via the submission site. Please log into your account, locate the manuscript record, and check for the action link "View Attachments". If this link does not appear, there are no attachment files.]

To ensure your figures meet our technical requirements, please review our figure guidelines: https://journals.plos.org/plosone/s/figures

You may also use PLOS’s free figure tool, NAAS, to help you prepare publication quality figures: https://journals.plos.org/plosone/s/figures#loc-tools-for-figure-preparation.

NAAS will assess whether your figures meet our technical requirements by comparing each figure against our figure specifications.

# Author comment: Author response to Decision Letter 1

**DOI:** 10.1371/journal.pone.0355181.r002

30 Jun 2026

Response to Reviewers

Manuscript ID: PONE-D-26-22703Title: Hyperhomocysteinemia in sudden sensorineural hearing loss: Evidence from a prospective clinical study

Dear Editor and Reviewers,

We would like to thank the Academic Editor and the reviewers for their careful evaluation of our manuscript and for their constructive comments. We have revised the manuscript accordingly and believe that the changes have substantially improved the clarity, methodological transparency, and scientific rigor of the study. All changes have been incorporated into the revised manuscript. Our point-by-point responses are provided below.

Editor / Reviewer comment 1:The Data Availability Statement should not state that data are available from the corresponding author. A non-author institutional contact should be provided.

Response:Thank you for this important comment. We have revised the Data Availability Statement accordingly. The revised statement now clarifies that the data are not publicly available due to ethical and institutional restrictions concerning participant confidentiality. We have removed the statement indicating that data are available from the corresponding author and instead provided an institutional contact point, namely the Afyonkarahisar Health Sciences University Clinical Research Ethics Committee or the relevant institutional data access body, for researchers who meet the criteria for access to confidential data.

Revised manuscript text:“The data underlying the findings of this study are not publicly available due to ethical and institutional restrictions concerning participant confidentiality. Data may be made available upon reasonable request from the Afyonkarahisar Health Sciences University Clinical Research Ethics Committee (contact number: +90 272 246 33 01) or the relevant institutional data access body, for researchers who meet the criteria for access to confidential data.”

Reviewer comment 2:The selection criteria and characteristics of the control group should be clarified.

Response:Thank you for this comment. We have revised the Materials and methods section to provide a clearer definition of the control group. We now specify that the control participants were age- and sex-matched individuals who presented to the internal medicine department for routine check-ups and had no history of sudden hearing loss, chronic otologic disease, vestibular schwannoma, chronic otitis media, autoimmune disease, ototoxic drug exposure, or active systemic infection. We also clarified that all control participants underwent otologic examination and pure tone audiometric evaluation to exclude clinically relevant hearing impairment.

Reviewer comment 3:The audiometric methodology should be revised to comply with standard audiometric procedures. Standard audiometry includes assessment from 250 Hz to 8 kHz and inter-octave frequencies when clinically indicated.

Response:Thank you for this important methodological comment. We agree with the reviewer and have revised the Audiological evaluation section. The revised text now states that pure tone audiometry was performed using standard air- and bone-conduction threshold measurements across conventional audiometric frequencies from 250 Hz to 8 kHz. We also clarified that additional inter-octave frequencies were assessed when clinically indicated, particularly when there was a threshold difference of 20 dB or more between two adjacent octave frequencies. For statistical analyses, the pure tone average was calculated using thresholds at 0.5, 1, 2, and 4 kHz.

Reviewer comment 4:The timing of the second blood sample should be clearly stated.

Response:Thank you for this comment. We have clarified the timing of the second venous blood sample in both the Abstract and the Laboratory analysis section. The revised manuscript now states that serum homocysteine levels were measured at baseline and at the 4–6-week follow-up visit after treatment in the SSNHL group, and once at enrollment in the control group.

Reviewer comment 5:The term “post-treatment” should not be used for the control group because controls did not receive treatment.

Response:Thank you for this important observation. We agree that this wording could be misleading. We have revised the Results section and Table 3 to clarify that the control group had only a single homocysteine measurement. We also added a table footnote stating that control group values represent a single homocysteine measurement because no treatment was administered to the control group.

Reviewer comment 6:The statistical reporting should follow AMA-style reporting standards, including appropriate descriptive statistics, test statistics, p-values, and effect sizes.

Response:Thank you for this valuable suggestion. We have revised the Statistical analysis section and the Results section accordingly. We assessed normality using the Shapiro–Wilk test and visual inspection of histograms and Q-Q plots. Normally distributed continuous variables were described as mean ± standard deviation, whereas non-normally distributed variables were presented as median and interquartile range. Categorical variables were presented as numbers and percentages. We also added the relevant test statistics and effect sizes for significant findings, including Mann–Whitney U values, Z values, p-values, and r effect sizes where appropriate. Logistic regression results are reported as odds ratios with 95% confidence intervals.

Reviewer comment 7:Effect sizes should be reported for significant findings.

Response:Thank you for this comment. We have added effect sizes for the significant non-parametric comparisons. For Mann–Whitney U and Wilcoxon signed-rank tests, effect size was calculated as r = Z/√N. These values are now presented in Table 3 and described in the Results section. For logistic regression, odds ratios with 95% confidence intervals are reported, and for ROC analysis, the area under the curve with 95% confidence interval is presented.

Reviewer comment 8:Table 5 contains values that appear unusually high and may represent incorrectly formatted PTA values.

Response:Thank you for pointing this out. We reviewed Table 5 and found that the values represented Spearman correlation coefficients, not PTA values. However, due to formatting, several correlation coefficients were displayed without decimal points, which may have caused confusion. We have corrected the table and now present each variable pair separately with Spearman’s rho and the corresponding p-value. The corrected values are reported as 0.261, 0.280, 0.085, and 0.227 rather than 261, 280, 85, and 227.

Reviewer comment 9:Table 8 should summarize all variables included in the logistic regression model, not only the significant predictor.

Response:Thank you for this comment. We have revised Table 8 to include all variables entered into the multivariate logistic regression model, including age, sex, diabetes mellitus, hypertension, coronary artery disease, and baseline homocysteine level. The revised table reports B, standard error, Wald statistic, odds ratio, 95% confidence interval, and p-value for each variable. The Results section has also been revised accordingly.

Reviewer comment 10:The SPSS version should be specified.

Response:Thank you for this comment. We have revised the Statistical analysis section to specify the software used. The revised manuscript now states: “All statistical analyses were performed using IBM SPSS Statistics for Windows, Version 26.0.”

Reviewer comment 11:Because the control group was age- and sex-matched, the manuscript should avoid interpreting age and sex as independent risk factors.

Response:Thank you for this important methodological point. We have revised the Discussion section to clarify that the study was not primarily designed to evaluate age or sex as independent risk factors for SSNHL because the control group was selected to be comparable with the SSNHL group in terms of age and sex. We also clarified that the absence of a significant association should not be interpreted as excluding the potential contribution of systemic vascular risk factors in SSNHL.

Reviewer comment 12:ROC curve analysis should be performed for baseline homocysteine levels.

Response:Thank you for this suggestion. We performed ROC curve analysis to evaluate the discriminative ability of baseline homocysteine levels for distinguishing SSNHL patients from controls. Baseline homocysteine demonstrated good discriminative ability, with an AUC of 0.809 (95% CI: 0.712–0.905, p < 0.001). The optimal cut-off value was 12.60 µmol/L, with a sensitivity of 75.0%, specificity of 85.0%, and Youden index of 0.600. These results have been added to the Results section, Table 9, and Fig 1. We also discussed the ROC findings in the Discussion section and emphasized that the cut-off value should be validated in larger independent cohorts before clinical application.

Reviewer comment 13:The Siegel classification may be limited when baseline contralateral hearing loss is present. How many patients had sensorineural hearing loss in the contralateral ear?

Response:Thank you for this important comment. We reviewed the baseline audiometric data of the unaffected contralateral ear. Clinically significant baseline contralateral sensorineural hearing loss was defined as a pure tone average greater than 25 dB in the unaffected ear at initial audiometric evaluation. Based on this definition, clinically significant baseline contralateral sensorineural hearing loss was present in 9 patients (22.5%). This information has been added to the Results section. We also acknowledged this issue in the Limitations section, noting that the Siegel classification may be influenced by pre-existing contralateral hearing loss and that this factor should be considered when interpreting treatment response.

Reviewer comment 14:Please disclose whether artificial intelligence tools or large language models were used during manuscript preparation.

Response:Thank you for this comment. We have added an Artificial Intelligence Disclosure section to the manuscript. The revised disclosure states that an AI-based language model was used only for language editing and improving clarity. No AI tool was used for study design, data collection, data analysis, interpretation of results, or generation of scientific conclusions. The authors reviewed and approved the final content and take full responsibility for the integrity and accuracy of the manuscript.

Reviewer comment 15:Please consider adding recent literature relevant to homocysteine, coagulation function, oxidative stress, and sudden deafness.

Response:Thank you for this helpful suggestion. We have added the recent study by Wang et al. concerning the relationship between coagulation function, homocysteine, serum oxidative stress, and sudden deafness. This reference has been incorporated into the Discussion section to strengthen the biological rationale linking homocysteine, oxidative stress, coagulation dysfunction, and cochlear microvascular impairment in SSNHL.

Reviewer comment 16:The manuscript should be revised according to PLOS ONE formatting requirements.

Response:Thank you for this comment. We revised the manuscript formatting in accordance with PLOS ONE style. The title has been changed to sentence case, section numbering has been removed, in-text citations have been changed from parentheses to square brackets, tables have been simplified, and the ROC curve has been presented as Fig 1 with an appropriate caption. We also revised the title page and declarations section to improve consistency with journal formatting requirements.

Reviewer comment 17:Please ensure that the manuscript includes appropriate ethical approval and informed consent information.

Response:Thank you for this comment. We have revised the Ethics Statement / IRB Approval / Informed Consent section. The manuscript now states that the study was conducted in accordance with the Declaration of Helsinki, that ethical approval was obtained from the Afyonkarahisar Health Sciences University Clinical Research Ethics Committee (Approval No: 2011-KAEK-2; Date: December 1, 2023), that institutional permission was obtained from the Department of Otorhinolaryngology, Faculty of Medicine, Afyonkarahisar Health Sciences University, and that written informed consent was obtained from all participants prior to enrollment.

We thank the Editor and reviewers again for their constructive comments. We believe that the revised manuscript now addresses all concerns raised during the review process.

Sincerely,

Murat AkınOn behalf of all authors

Attachment Submitted filename: Response to Reviewers.docx

# Peer review: Decision Letter 1

**DOI:** 10.1371/journal.pone.0355181.r003

**Authors:** Gauri Mankekar

19 Jul 2026

<p>Hyperhomocysteinemia in Sudden Sensorineural Hearing Loss: Evidence From a Prospective Clinical Study

PONE-D-26-22703R1

Dear Dr. Murat Akın,

We’re pleased to inform you that your manuscript has been judged scientifically suitable for publication and will be formally accepted for publication once it meets all outstanding technical requirements.

Within one week, you’ll receive an e-mail detailing the required amendments. When these have been addressed, you’ll receive a formal acceptance letter and your manuscript will be scheduled for publication.

An invoice will be generated when your article is formally accepted. Please note, if your institution has a publishing partnership with PLOS and your article meets the relevant criteria, all or part of your publication costs will be covered. Please make sure your user information is up-to-date by logging into Editorial Manager at Editorial Manager® and clicking the ‘Update My Information' link at the top of the page. For questions related to billing, please contact billing support.

If your institution or institutions have a press office, please notify them about your upcoming paper to help maximize its impact. If they’ll be preparing press materials, please inform our press team as soon as possible -- no later than 48 hours after receiving the formal acceptance. Your manuscript will remain under strict press embargo until 2 pm Eastern Time on the date of publication. For more information, please contact onepress@plos.org.

Kind regards,

Gauri Mankekar, MD,PhD,FACS

Academic Editor

PLOS One

Additional Editor Comments (optional):

Reviewers' comments:

Reviewer's Responses to Questions

**Comments to the Author**

1. If the authors have adequately addressed your comments raised in a previous round of review and you feel that this manuscript is now acceptable for publication, you may indicate that here to bypass the “Comments to the Author” section, enter your conflict of interest statement in the “Confidential to Editor” section, and submit your "Accept" recommendation.

Reviewer #1: All comments have been addressed

Reviewer #2: (No Response)

**********

2. Is the manuscript technically sound, and do the data support the conclusions?

The manuscript must describe a technically sound piece of scientific research with data that supports the conclusions. Experiments must have been conducted rigorously, with appropriate controls, replication, and sample sizes. The conclusions must be drawn appropriately based on the data presented.

Reviewer #1: Yes

Reviewer #2: Yes

**********

3. Has the statistical analysis been performed appropriately and rigorously?

Reviewer #1: Yes

Reviewer #2: Yes

**********

4. Have the authors made all data underlying the findings in their manuscript fully available?

The PLOS Data policy requires authors to make all data underlying the findings described in their manuscript fully available without restriction, with rare exception (please refer to the Data Availability Statement in the manuscript PDF file). The data should be provided as part of the manuscript or its supporting information, or deposited to a public repository. For example, in addition to summary statistics, the data points behind means, medians and variance measures should be available. If there are restrictions on publicly sharing data—e.g. participant privacy or use of data from a third party—those must be specified.

Reviewer #1: Yes

Reviewer #2: Yes

**********

5. Is the manuscript presented in an intelligible fashion and written in standard English?

PLOS ONE does not copyedit accepted manuscripts, so the language in submitted articles must be clear, correct, and unambiguous. Any typographical or grammatical errors should be corrected at revision, so please note any specific errors here.

Reviewer #1: Yes

Reviewer #2: Yes

**********

6. Review Comments to the Author

Please use the space provided to explain your answers to the questions above. You may also include additional comments for the author, including concerns about dual publication, research ethics, or publication ethics. (Please upload your review as an attachment if it exceeds 20,000 characters)

Reviewer #1: Thanks for the opportunity to review your manuscript. I beleive the manuscript is in a good shape after addressing all the comments from the editor and the reviewers.

Reviewer #2: I again recommend against using an asterisk in the tables (Table 3), since this is often used to mean a statistical significance. The authors have otherwise addressed my concerns.

**********

7. PLOS authors have the option to publish the peer review history of their article (what does this mean?). If published, this will include your full peer review and any attached files.

If you choose “no”, your identity will remain anonymous but your review may still be made public.

**Do you want your identity to be public for this peer review?**  For information about this choice, including consent withdrawal, please see our Privacy Policy.

Reviewer #1: No

Reviewer #2: No

**********

# Editor report: Acceptance letter

**DOI:** 10.1371/journal.pone.0355181.r004

**Authors:** Gauri Mankekar

PONE-D-26-22703R1

PLOS One

Dear Dr. Akın,

I'm pleased to inform you that your manuscript has been deemed suitable for publication in PLOS One. Congratulations! Your manuscript is now being handed over to our production team.

At this stage, our production department will prepare your paper for publication. This includes ensuring the following:

* All references, tables, and figures are properly cited

* All relevant supporting information is included in the manuscript submission,

* There are no issues that prevent the paper from being properly typeset

You will receive further instructions from the production team, including instructions on how to review your proof when it is ready. Please keep in mind that we are working through a large volume of accepted articles, so please give us a few days to review your paper and let you know the next and final steps.

Lastly, if your institution or institutions have a press office, please let them know about your upcoming paper now to help maximize its impact. If they'll be preparing press materials, please inform our press team within the next 48 hours. Your manuscript will remain under strict press embargo until 2 pm Eastern Time on the date of publication. For more information, please contact onepress@plos.org.

You will receive an invoice from PLOS for your publication fee after your manuscript has reached the completed accept phase. If you receive an email requesting payment before acceptance or for any other service, this may be a phishing scheme. Learn how to identify phishing emails and protect your accounts at https://explore.plos.org/phishing.

If we can help with anything else, please email us at customercare@plos.org.

Thank you for submitting your work to PLOS ONE and supporting open access.

Kind regards,

PLOS ONE Editorial Office Staff

on behalf of

Dr. Gauri Mankekar

Academic Editor

PLOS One
